# Role of KIR and CD16A genotypes in colorectal carcinoma genetic risk and clinical stage

**DOI:** 10.1186/s12967-016-1001-y

**Published:** 2016-08-12

**Authors:** Angelica Canossi, Anna Aureli, Tiziana Del Beato, Piero Rossi, Luana Franceschilli, Flavio De Sanctis, Pierpaolo Sileri, Nicola di Lorenzo, Oreste Buonomo, Davide Lauro, Adriano Venditti, Giuseppe Sconocchia

**Affiliations:** 1 Laboratory of Tumor Immunology and Immunotherapy, CNR Institute of Translational Pharmacology (IFT), Via Fosso del Cavaliere 100, 00133 L’Aquila, Rome, Italy; 2 Department of Experimental Medicine and Surgery, University of Rome Tor Vergata, Rome, Italy; 3 Department of Systems Medicine, University of Rome Tor Vergata, Rome, Italy; 4 Department of Biomedicine and Prevention, University of Rome Tor Vergata, Rome, Italy

**Keywords:** NK, KIR, CD16A, FCGR3A genotypes, Colorectal carcinoma, CRC, Genetic risk

## Abstract

**Background:**

NK cell cytotoxicity is regulated by the types of the interaction between killer immunoglobulin-like receptors (KIRs) and human leukocyte antigen (HLA) class I ligands on target cells and the different binding affinity of the Fcγ receptor IIIA (CD16A) for IgG-coated tumor cells. Thus, it is conceivable that KIR and CD16A gene contents may contribute to the function of NK cells by modulating an immune response in the colorectal carcinoma (CRC) microenvironment. This hypothesis is supported by recent evidence suggesting that NK cells improve the clinical course of CRC patients by enhancing the anti-CRC effect of CD8 + T cells. This information provides the rationale to test the hypothesis whether the independent KIR segregation and specificity, as well as CD16A gene polymorphisms, have an impact on CRC.

**Methods:**

Using polymerase chain reaction-sequence-specific primers (PCR-SSP) and sequence-based typing (SBT), we investigated KIR/HLA-C complex and CD16A (48H/R/L,158V/F) gene polymorphisms in 52 CRC patients and 61 local healthy controls (LCTRs).

**Results:**

The allele frequency (AF) of at least five activating KIR (aKIRs) of the B haplotype (p = 0.036, OR 0.204), KIR2DL2 (p = 0.047, OR 0.2616), and KIR2DS2 genes (5.8 vs LCTR 13.8 % and vs. Fasano’s CTR 16.3 %, p = 0.05, OR 0.3145), in the absence of their cognate HLA-C1 ligands, were significantly associated with a reduced genetic risk of CRC. In contrast, CD16A-48H polymorphism was positively associated with an increased genetic risk of CRC (p = 0.05, OR 2.761). The latter was also found to be correlated with advanced stages of disease [III and IV (p = 0.03, OR 3.625)].

**Conclusions:**

Our data suggest that the analysis of aKIRs and KIR2DL2 gene and CD16A-48H may be of interest for the identification of individuals at reduced and increased genetic risk of CRC, respectively.

**Electronic supplementary material:**

The online version of this article (doi:10.1186/s12967-016-1001-y) contains supplementary material, which is available to authorized users.

## Background

Compelling evidence suggests that a pro-inflammatory solid tumor microenvironment composed of CD8 + T lymphocytes is associated with a favorable course of disease [1–3]. In contrast, a solid tumor microenvironment infiltrated by anti-inflammatory cells including tumor-associated macrophages (TAMs) [4] and regulatory T cells (Tregs) [5] is an unfavorable prognostic factor, and it is associated with poor prognosis. However, CRC seems to be an exception since inflammation, regardless the type of cellular infiltration including TAMs [6–9] and Tregs [10], is associated with a favorable prognosis. In this context, however, the role of NK cells is still debated [11, 12]. The argument involving the role of NK cells in CRC is in part due to the narrowed degree of NK cell infiltration of solid tumors [12]. The reduced NK cell infiltration in the tumor bed can be, at least in part, explained by the detrimental effect of malignant cells on NK cells. Besides, there is also conflicting information about the clinical relevance of NK cells in CRC [13–15]. Recent findings, however, suggest that intra-tumor NK cell infiltration improves the clinical outcome of CRC by enhancing the protective role of tumor-infiltrating CD8 + T cells [16]. In addition, loss or down-regulation of HLA class I molecules on autologous CRC cells promotes NK cell cytotoxicity [17]. Because KIRs are independently segregated, an autologous KIR-HLA mismatched may exist. As a consequence, NK cells can trigger an enhanced cytotoxicity, as compared with NK cells expressing self-recognizing inhibitory KIRs [18].

KIRs consist of immunoglobulin-like receptors and heterodimers [19]. Immunoglobulin-like KIRs, with a long intracellular tail (KIRL), mediate inhibitory activity while those with a short intracellular tail (KIRS) can function as activating molecules. The HLA-C antigens are the best-known KIR ligands. Based on polymorphisms at positions 77 and 80, in the α1-domain of the β chain, KIRs can be identified into two additional groups: the HLA-C1 that carries an asparagine residue at position 80, and the HLA-C2 that has a lysine residue at position 80 [20, 21]. To date, KIR genotyping is utilized for the analysis of gene content and categorization of A/B haplotypes as well as for prediction of NK cell reactivity in autologous and allo NK cell-based immunotherapy [22, 23]. The A haplotype contains several inhibitory KIR genes and only one activating KIR. Conversely, B haplotype displays more activating KIR genes. Based on the linkage disequilibrium between particular alleles of different KIR loci, within the B haplotype, two gene clusters are identified. The first group consists of four centromeric KIR genes (C4 group), while the second group contains telomeric genes (T4 group) [24]. Recently, several studies have indicated a relationship between KIR genotype and cancer, but there is scant information about a link between KIR genes and CRC [25–27].

A primary mechanism by which NK cells kill CRC cells is the antibody-dependent cellular cytotoxicity (ADCC). This function is a result of the engagement of the Fc fragment of an antibody to the CD16A antigen on NK cell surface. The extent of ADCC relies on the binding affinity of the Fc fragment of the mAb to CD16A that is in turn regulated by CD16A polymorphisms [28]. In fact, the presence of valine/valine (V/V), but not valine/phenylalanine (V/F) at position 158, enhances ADCC and human IgG1 binding to CD16A [29]. Among monoclonal antibodies (mAbs), the anti-epidermal-growth factor receptor (EGFR), namely cetuximab has been successfully utilized for targeting therapy of EGFR+ malignancies including CRC [30]. Furthermore, cetuximab -activated NK cells have been shown to exert an indirect anti-tumor effect by promoting tumor antigen-anti-EGFR-specific T cells [31]. Thus, the genetic distribution of KIRs and CD16A is likely to have a substantial impact on NK cell phenotype and function that may affect the CRC pathogenesis. This information provides the rationale for testing the effect of KIR and CD16A genotypes on the genetic susceptibility to develop CRC and some clinical features.

## Methods

### Patient and control samples

The case–control study was performed using peripheral blood cells obtained from Italian CRC patients, recruited at the Policlinic of Tor Vergata between August 2010 and December 2012. This study (protocol number: 133/10) was approved by an independent, Institutional Review Board (IRB) Committee at the Teaching Hospital of Tor Vergata, Rome, Italy. Fifty-two CRC patients (31 males and 21 females), aged between 32 and 88 years, without a previous history of inflammatory bowel disease (IBD) and autoimmune diseases, were included in this protocol. Staging of the disease was assessed according to the pathological classification of tumor-node-metastasis (pTNM), as shown in Table 1. Control population comprised 61 healthy subjects (26 males and 35 females) originating from CRC patients geographical area, hereafter referred to as local controls (LCTRs) and historical controls [32, 33].

**Table 1:** Demographic and clinical characteristics of 52 CRC patients (from Central Italy)

Gender n (%)	Count, frequency (%)
Male	31 (59.6)
Female	21 (40.4)
Age average (years) ± standard deviation	
All patients	70.8 ± 11.1
Male	74.1 ± 7.85
Female	66.2 ± 13.63
Clinical tumor stage, %	
I	15 (28.8)
II	16 (30.8)
III	15 (28.8)
IV	4 (7.7)
Unknown	2 (3.8)
Location, %	
Ascending right colon	19 (36.5)
Transverse colon	3 (5.8)
Descending left colon	10 (19.2)
Cecum	1 (1.9)
Rectum	19 (36.5)
Unknown	1 (1.9)

### DNA extraction and KIR genotyping

Peripheral blood cell DNA was isolated using spin bind columns (QIAmp Blood Midi kit, *Qiagen*), which purify genomic DNA from contaminants and proteins. KIR genotyping was performed using PCR-SSP primers for 17 KIR genes (KIR2DL1, 2DL2, 2DL3, 2DL4, 2DL5A, 2DL5B, 2DS1, 2DS2, 2DS3, 2DS4del and 2DS4ins, 2DS5, 3DL1, 3DL2, 3DL3, 3DS1 and 2DP1, 3DP1 pseudogenes) (*Miltenyi Biotech*). PCR products were visualized after a 2 % gel electrophoresis containing ethidium bromide and photo-documented on a UV trans-illuminator. KIR haplotypes were classified as previously reported [34].

### HLA-C allele genotyping

HLA-C typing was performed by using a sequence-based typing (SBT) kit with a bigdye terminator chemistry v.1.1 (AlleleSEQR HLA-C kit, Atria) which analyzes the allelic polymorphisms in exons 2-4 of HLA-C gene. Different HLA-C alleles were divided in C1(HLA-C*01, 03,07,08,09,10,12,14) and C2 (HLA-C*02,04,05,06,15) groups, depending on the presence of Asparagine or Lysine at position 80, respectively. The study of the interactions between a specific KIR and subsets of HLA-C allotypes (C1 and C2) was established according to the *Campbell* KS’s review scheme [35].

### CD16A genotyping by sequence-based typing

The genotyping of CD16A-158V/F and CD16A-48 leucine (L)/arginine (R)/histidine (H) was performed on genomic DNA by polymerase chain reaction (PCR) using primers previously described [36]. Briefly, PCR reactions were set up with 50 ng of genomic DNA per 50 µl reaction and amplified products were purified and sequenced using Big Dye Terminator Chemistry (Applied Biosystems, Foster City, CA) on an ABI Prism 3130 Genetic Analyzer (PE Applied Biosystems). Sequencing reactions were purified, and the analysis of results was obtained by alignment of the processed sequences and the reference human CD16A gene.

### Statistical analysis

Pearson X^2^ test was used for comparison of the gene frequency of the patient group with controls. Fisher’s exact test was used when the expected difference between the two groups was small. The odds ratio (OR) and its 95 % confidence intervals (CI) were calculated. A *p* value of 0.05 or less was considered to be significant. The influence of KIR genes with or without association with their HLA-C ligands was assessed using the binary logistic regression analysis (IBM SPSS Statistics v.19). The SPSS statistical package, Version 19 for Windows, was used for data and statistical analysis. CD16A haplotype frequencies were assessed by an expectation-maximum (EM) algorithm. Thus, samples containing one allele were considered homozygous, and that allele was counted twice in the analysis. For multilocus genotypic data, the maximum likelihood was estimated using an EM algorithm when the gametic phase was not known. To determine whether the observed diploid genotypes were the product of a random gamete union, the Hardy–Weinberg equilibrium was calculated by the Guo and Thomson exact test [37]. The analysis of molecular polymorphisms at CD16A-48 and 158, within the population, was performed using the ARLEQUIN V.3.0 POPULATION GENETICS software.

## Results

### Association of five aKIRs of the B haplotype with a reduced susceptibility to CRC

The extent of KIR gene differences in 52 CRC patients and controls was assessed by SSP genotyping of 16 different NK receptor genes (data provided in Additional file 1: Table S1). To determine whether a distinct KIR haplotype frequency is associated with a reduced or increased risk for a genetic susceptibility to CRC, we compared the A and B haplotype frequencies of CRC patients with those of LCTRs. In both sample groups, the A/B haplotypes were the most represented (LCTR 70.5 % and CRC 73.1 %). Within the B haplotype, we considered different centromeric and telomeric genes, and we noted a reduced percentage of the C4T4 subtypes in CRC patients as compared to LCTR (5.8 vs. 16.4 %, Additional file 2: Table S2). More importantly, we found a significantly increased frequency of aKIR genes (≥5) in the B haplotype of LCTRs than that of CRC patients (16.4 vs. 3.9 %, p = 0.036, OR 0.204). These results were further validated when the frequency of aKIRs in the B haplotype of Italian historical controls [32] was compared to that of CRC patients (p = 0.0005), Table 2. These data suggest that the presence of ≥5 aKIR genes may protect healthy subjects from CRC.

**Table 2:** Activating KIR frequency in CRC patients and Italian controls

aKIRs	CRC pts (n = 52)		Local CTRS (n = 61)		p value	Caggiari CTRS (n = 69)		p value
	N	%	N	%		N	%	
4	16	30.8	24	39.3	NS	19	27.5	NS
5–6	2	3.9	10	16.4	0.036	19	27.5	0.0005

### Enhanced KIR2DL2 and KIR2DS2 allele frequencies, in the absence of their HLA-C ligand, is associated with a reduced susceptibility to CRC

Since the NK receptor/HLA ligand interaction controls NK-cell function influencing both the licensing and intensity of the NK cell immune response, we examined such a combination in CRC patients and controls by their cognate HLA-C ligand. We found that both frequencies of single HLA-C alleles and groups of KIRs, categorized according to the allelic residues at position 80 (Asn/Lys), were not significantly different between CRC patients and controls. Nevertheless, there was a statistically positive trend toward an increased frequency of HLA-C1 alleles Asn80 in CRC patients over LCTRs (59.6 vs. 47.4 %, p = 0.07), Additional file 3: Table S3.

We then examined the frequency of KIR2DL1, KIR2DL2, KIR2DL3 and their corresponding aKIRs in the presence or absence of their HLA-C ligands. We found a significantly lower incidence of KIRDL2 gene (5.8 vs. LCTR 19.0 %, p = 0.04, OR 0.2616) and KIR2DS2 gene, (5.8 vs 13.8 % and vs. Fasano’s CTR 16.3 %, p = 0.05, OR 0.3145), in the absence of their cognate HLA-C1 ligand, in CRC patients compared to local and historical controls [33]. These results suggest that the presence of KIR2DL2 and KIR2DS2, in the absence of their respective HLA C1 ligands, may protect one from the disease (Table 3). The KIR2DS2 results were further supported by a binary logistic regression analysis (p = 0.008, OR  0.186).

**Table 3:** Distribution of KIR/HLA-C combinations in CRC patients, local healthy samples and Italian controls

KIR genes and HLA ligands	CRC patients (N = 52)		Local controls (N = 58)		p value^a^	OR	Fasano, 2014 (N = 270)		p value^a^	OR
	N	%	N	%			N	%		
2DL1 HLA-C2+	34	65.4	43	74.1	NS		187	69.3	NS	
2DL1 HLA-C2−	17	32.7	13	22.4	NS		74	27.4	NS	
2DL2 HLA-C1+	28	53.8	26	44.8	NS		113	41.9	NS	
2DL2 HLA-C1/C1	11	21.2	5	8.6	NS					
2DL2 HLA-C1−	3	5.8	11	19.0	0.047	0.2616	43	16.0	0.08	
2DL3 HLA-C1+	40	76.9	35	60.3	NS		183	67.8	NS	
2DL3 HLA-C1−	6	11.5	11	19.0	NS		51	18.8	NS	
2DS1 HLA-C2+	13	25.0	22	37.9	NS		68	25.2	NS	
2DS1 HLA-C2−	7	13.5	6	10.3	NS		29	10.7	NS	
2DS2 HLA-C1+	30	57.7	24	41.4	NS		112	41.5	0.033	1.9237
2DS2 HLA-C1−	3	5.8	8	13.8	NS		44	16.3	0.05	0.3145
2DS2 AND 2DS3 HLA-C1+	16	30.8	14	24.1	NS					
2DS2+ , KIR2DL2 or 2DL3+ , C group 1+	30	57.7	23	39.7	0.08					
2DL2 and 2DS2+ , C group 1 −	2	3.8	8	13. 8	0.09					

^a^Pearson Chi square test or Fisher’s exact test, as appropriate

### The impact of CD16A-48 allele variants on CRC genetic susceptibility and CRC pathological staging

To determine the role of CD16A genotypes in the genetic susceptibility to CRC and pathological stage, we compared the 559G/T (158, V/F) and 230A/G/T (48, H/R/L) allele polymorphisms in CRC patients and LCTRs (Table 4). CD16A-48H was significantly associated with CRC (p = 0.05, OR  2.761) and stages III–IV (p = 0.03, OR  3.625). Considering CD16A allele polymorphisms in haplotype (Table 5), four CD16A-48/158 haplotypes (48T/158G, 48T/158T, 48A/158G, 48G/158G) were assessed. CD16A-48T/158G and 48T/158T haplotypes were the best represented (i.e. 42.6 and 41.0 % in LCTR) and CD16A-48H being completely linked to 158 V in CRC patients and LCTRs.

**Table 4:** Distribution of CD16A-48 and -158 genetic polymorphisms in Italian local controls and CRC patients

Genotype^a^	LCTRS (n = 61) N (%)	CRC (n = 52) N (%)	p value	OR	Grade I–II (n = 31) N (%)	P value	Grade III–IV (n = 19) N (%)	p value	OR
AA158									
CD16A-559T/T	5 (8.2)	4 (7.7)	NS	NS	2 (6.5)	NS	2 (10.5)	NS	NS
CD16A -559G/T	40 (65.6)	32 (61.5)	NS	NS	20 (64.5)	NS	11 (57.9)	NS	NS
CD16A -559G/G	16 (26.2)	16 (30.8)	NS	NS	9 (29.0)	NS	6 (31.6)	NS	NS
CD16A -559GT + TT	47 (74.6)	36 (69.2)	NS	NS	22 (71.0)	NS	13 (68.4)	NS	NS
Allele									
CD16A -V158 (G)	72 (59.0)	64 (61.5)	NS	NS	38 (61.3)	NS	23 (60.5)	NS	NS
CD16A -F158 (T)	50 (41.0)	40 (38.5)	NS	NS	24 (38.7)	NS	15 (39.5)	NS	NS
AA48									
CD16A -230A/G	0	1 (1.9)	NS	NS	0	NS	1 (5.3)	NS	NS
CD16A -230G/T	10 (16.4)	5 (9.6)	NS	NS	2 (6.5)	NS	3 (15.8)	NS	NS
CD16A -230G/G	2 (3.3)	0	NS	NS	0	NS	0	NS	NS
CD16A -230T/T	43 (70.5)	34 (65.4)	NS	NS	21 (67.7)	NS	11 (57.9)	NS	NS
CD16A -230A/T^a^	6 (9.8)	12 (23.1)	0.07	NS	7 (22.6)	NS	5 (26.3)	NS	NS
Allele									
CD16A -H48 (A)^a^	6 (4.9)	13 (12.5)	0.05	2.761	7 (11.3)	NS	6 (15.8)	0.037	3.625
CD16A -R48 (G)	14 (11.5)	6 (5.8)	NS		3 (4.8)	NS	3 (7.9)	NS	
CD16A -L48 (T)	102 (83.6)	85 (81.7)	NS		52 (83.9)	NS	29 (76.3)	NS	

^a^No deviation from Hardy–Weinberg equilibrium was observed for the 559 and 230 allelic polymorphisms

**Table 5:** Distribution of CD16A-48 and -158 haplotype frequencies in Italian LCTR and CRC patients, also distinguished for tumor grading

CD16A Haplotype	LCTR HF (n = 61)	CRC HF (n = 52)	p value^b^	Grade I–II^a^ (n = 31) (af %)	P value^b^	Grade III–IV^a^(n = 19) (af %)	p value^b^
48T-158G (48L158 V)	0.42623 ± 0.045824	0.432692 ± 0.049342	NS	45.2	NS	36.8	NS
48T-158T (48L158F)	0.409836 ± 0.047115	0.384615 ± 0.047437	NS	38.7	NS	39.5	NS
48A-158G (48H158 V)	0.049180 ± 0.01791	0.12500 ± 0.033282	0.05^^^	11.3	NS	15.8	0.037^§^
48G-158G (48R158 V)	0.114754 ± 0.026747	0.057692 ± 0.02222	NS	4.8	NS	7.9	NS

^§^ CD16-48A/158G OR 3.625

^ OR 2.7619

^a^Patients with known staging included in the study (N = 50)

^b^Pearson X^2^ test: FCGR3A-48A/158G vs 48G/158G haplotype *p* = *0.0256*

## Discussion

Our study strengthens the protective role of NK cells in CRC that is supported by our KIR results in which a prevalence of the B haplotype hosting ≥5 aKIR genes, KIR2DS2 and KIR2DL2, in the absence of their HLA-C ligands, protects one from developing CRC. On the contrary, the contribution of NK cells to the CD16A-48H genotype-increased susceptibility to CRC and its association with advanced stages of disease is not readily apparent.

It is noteworthy that most of the studies addressing the role of NK cells in CRC have been performed utilizing immunohistochemistry. This methodology provides relevant information about the qualitative and quantitative aspects of NK cell infiltration in the CRC microenvironment which can be correlated with the clinical course of disease. However, it cannot be utilized to identify healthy subjects at risk of developing disease. To this end, KIR genotyping may provide useful information about the possibility to determine a healthy subject at genetic risk of CRC.

In this study, we have compared the KIR genotype allele frequency of CRC patients to that of healthy local and historical controls, expecting to find a reduced allele frequency of activating KIRs and inhibitory KIRs, in the absence of their HLA-C ligands in CRC patients. In support of our hypothesis, we found a significantly reduced frequency of ≥5 aKIRs, belonging to the B haplotype, in CRC patients when compared to LTCRs. In agreement with our study, other additional scientific reports, performed by KIR genotyping of CRC patients, have demonstrated an association of multiple aKIRs with long-term disease-free survival [38] and favorable responses to chemotherapy in patients with metastatic CRC [27]. An obvious interpretation of these data is that the presence of multiple KIRs may overcome the inhibitory effects of the KIRs on NK cells increasing the level of NK cell activation. On the other hand, it has been shown that NK cells are capable of eliminating autologous CRC cells [39]. According to the missing self-hypothesis, an enhanced level of NK cell activation may also be applied to the interpretation of the protective effect of KIR2DL2 in healthy subjects, lacking their HLA-C1 ligand genes. In contrast, the analysis of the significance of KIR2DS2 is harder to comprehend since it is not clear whether KIR2DS2 can activate NK cells in the absence of their HLA-C1 ligands.

The unique protective role of KIR2DL2 over the other KIRs may rely on the fact that KIR2DL2 is one of the most common KIR, nearly present in all individuals. Moreover, KIR2DL2 is also expressed by NK-T lymphocytes leading to an increased number of effector cells with anti-CRC potential [40]. The protective role of KIR2DL2, in the absence of the HLA-C1 ligands, is not restricted to CRC but is also demonstrated in inflammatory bowel disease, including Crohn’s disease [41], underlying the importance of this KIR in NK cell immunity. In contrast, it is quite difficult to distinguish the role of KIR2DS2 from that of KIR2DL2. Considering the protective effect is achieved in the absence of HLA-C1 ligands, while an active KIR2DS2 needs to bind the HLA-C1 ligands, there is a chance that this effect may not be related to KIR2DS2 but is rather restricted to the influence of KIR2DL2 gene [42].

The complexity of this topic is underlined by the evidence that the presence of ≥5 aKIRs is associated with human papilloma virus-induced cervical neoplasia [43]. Furthermore, a second study has shown that the presence of an increased number of aKIR genes is also found to be associated with the Epstein-Barr virus- linked nasopharyngeal carcinoma (NPC) [44].

Thus, the presence of ≥5 activating KIRs has a different effect in solid tumors being protective in CRC and predisposing in cervical and nasopharyngeal carcinomas. One can speculate that this difference may reflect the possibility that NK cells are involved in the generation of two types of inflammation with opposite effects on tumor growth and development. In this context, NK cells may enhance the anti-CRC effect of CD8 + T cells by releasing interferon γ which up-regulates the expression of the HLA-class I and class II on the surface of CRC cells [45]. In contrast, NK cell activation, following virus-induced malignancies, such as EBV or HPV, may trigger mechanism(s) of tumor immunoevasion from the NK cell immune surveillance. On the other hand, it has been clearly shown that NK cells can damage dendritic cells and macrophages, both involved in the presentation of tumor-associated antigen(s) to T cells [46].

The extent of ADCC mediated by CD16A, in the presence of mAbs, depends on the cell types including NK cells, monocytes, and granulocytes and CD16A polymorphisms. This is because the latter possesses the ability to bind the Fc fragment of mAbs with different affinity, affecting the extent of ADCC against tumor cells [47, 48]. Then, guided by recent findings indicating that some CD16A polymorphisms predict the therapeutic effectiveness of cetuximab in EGFR+ cancer patients [49], we found that only CD16A-48H gene variant is involved, being more associated with CRC patients and advanced stages of CRC. This association may reflect a status of immunodeficiency in CRC patients since the presence of CD16A-48H gene is linked to patients with episodes of recurrent viral infection of the respiratory tract supporting an in vivo NK cell abnormality [50]. By the available data, it is not possible to identify a cause/effect relationship between CD16A-48H phenotype and NK cell immunodeficiency. However, the presence of CD16A-48H results in a loss of the Leu11c/B73.1 epitope on NK cells that leads to the inhibition of the IgG binding to the CD16A [51, 52]. In this light, we speculate that CD16A-CD48H may favor CRC development by an impaired ADCC against CRC cells.

## Conclusions

Our study sustains the possibility that aKIR genotyping of the B haplotype, KIR2DL2 and HLA-C1 may be utilized to identify healthy individuals at reduced risk of developing CRC, while the genotyping of the CD16A-158V/48H haplotype is of interest for the identification of those at increased risk of CRC and tumor staging.

## Additional files

10.1186/s12967-016-1001-y KIR gene frequencies in CRC patients in comparison with local controls.
10.1186/s12967-016-1001-y A and B haplotypes frequencies and B subtypes in CRC patients and healthy controls.
10.1186/s12967-016-1001-y Allele frequencies of HLA-C types (C1 R80 and C2 K80) in CRC patients and Italian controls.

## Abbreviations

KIR: killer immunoglobulin-like receptors
NK: natural killer
HLA: human leukocyte antigen
CD16a: cluster of differentiation 16A
CD8: cluster of differentiation 8
CRC: colorectal carcinoma
PCR-SSP: polymerase chain reaction-sequence-specific primers
SBT: sequence-based typing
LCTRs: local healthy controls
AF: allele frequency
aKIR: activating KIR
TAM: tumor-associated macrophages
Treg: regulatory T cells
ADCC: antibody-dependent cellular cytotoxicity
