# Supplementary material for: Role of KIR and CD16A genotypes in colorectal carcinoma genetic risk and clinical stage
Source: J Transl Med. 2016 Aug 12;14:239. doi: 10.1186/s12967-016-1001-y (PMC4983069; doi:10.1186/s12967-016-1001-y)
Supplement: Supplementary file 1 — 10.1186/s12967-016-1001-y KIR gene frequencies in CRC patients in comparison with local controls. [file 12967_2016_1001_MOESM1_ESM.docx]

**Table S1 - KIR gene frequencies (%) in CRC patients (n=52) in comparison with local controls (n=61).**

| **KIR GENE** | **COLORECTAL CANCER**  **PATIENTS**  **(N=52)** | |  | **LOCAL CONTROLS**  **(N=61)** | | **P-value *** |
| --- | --- | --- | --- | --- | --- | --- |
|  | **N** | **%** |  | **N** | **%** |  |
| **2DL1** | 51 | 98.1 |  | 59 | 96.7 | NS |
| **2DL2** | 31 | 59.6 |  | 37 | 60.7 | NS |
| **2DL3** | 46 | 88.5 |  | 49 | 80.3 | NS |
| **2DL4** | 52 | 100.0 |  | 61 | 100.0 | NS |
| **2DL5A** | 21 | 40.4 |  | 27 | 44.3 | NS |
| **2DL5B** | 26 | 50.0 |  | 37 | 60.7 | NS |
| **2DS1** | 20 | 38.5 |  | 28 | 45.9 | NS |
| **2DS2** | 33 | 63.5 |  | 33 | 54.1 | NS |
| **2DS3** | 16 | 30.8 |  | 22 | 36.1 | NS |
| **2DS4del *003** | 46 | 88.5 |  | 47 | 77.0 | NS |
| **2DS4ins *001-002** | 17 | 32.7 |  | 22 | 36.1 | NS |
| **2DS5** | 14 | 26.9 |  | 21 | 34.4 | NS |
| **3DL1** | 47 | 90.4 |  | 55 | 90.2 | NS |
| **3DL2** | 52 | 100.0 |  | 61 | 100 | NS |
| **3DL3** | 52 | 100.0 |  | 61 | 100 | NS |
| **3DS1** | 21 | 40.4 |  | 28 | 45.9 | NS |

*Pearson Chi-Square test or Fisher’s Exact test, as appropriate.
