# Supplementary material for: Role of KIR and CD16A genotypes in colorectal carcinoma genetic risk and clinical stage
Source: J Transl Med. 2016 Aug 12;14:239. doi: 10.1186/s12967-016-1001-y (PMC4983069; doi:10.1186/s12967-016-1001-y)
Supplement: Supplementary file 2 — 10.1186/s12967-016-1001-y A and B haplotypes frequencies and B subtypes in CRC patients and healthy controls. [file 12967_2016_1001_MOESM2_ESM.docx]

**Table S2 – Haplotypes A and B frequencies and B subtypes in CRC patients and healthy controls.**

| **KIR haplotypes** | **CRC**  **(n=52)** | | **Caggiari, 2011**  **(n=69)** | | **P-value** | **local ctrs**  **(n=61)** | | **P-value** |
| --- | --- | --- | --- | --- | --- | --- | --- | --- |
|  | **N** | **%** | **N** | **%** |  | **N** | **%** |  |
| AA | 13 | 25.0 | 25 | 36.2 | NS | 16 | 26.2 | NS |
| **AB** | 38 | 73.1 | 42 | 60.9 | NS | 43 | 70.5 | NS |
| BB | 1 | 1.9 | 2 | 2.9 | NS | 2 | 3.3 | NS |
| **C4T4** | **3** | **5.8** | 7 | 10.1 | NS | **10** | **16.4** | NS |
| C4TX | 11 | 21.2 | 10 | 14.5 | NS | 10 | 16.4 | NS |
| CXT4 | 11 | 21.2 | 12 | 17.4 | NS | 7 | 11.5 | NS |
| CXTX | 14 | 26.9 | 15 | 21.7 | NS | 19 | 31.1 | NS |

*Pearson Chi-Square test or Fisher’s Exact test, as appropriate. CRC: Haplo A : 64/104=61.5%, Haplo B: 40/104=38.5% LCTRS: Haplo A 75/122=61.5% Haplo B 47/122=38.5%
