# Supplementary material for: Role of KIR and CD16A genotypes in colorectal carcinoma genetic risk and clinical stage
Source: J Transl Med. 2016 Aug 12;14:239. doi: 10.1186/s12967-016-1001-y (PMC4983069; doi:10.1186/s12967-016-1001-y)
Supplement: Supplementary file 3 — 10.1186/s12967-016-1001-y Allele frequencies of HLA-C types (C1 R80 and C2 K80) in CRC patients and Italian controls. [file 12967_2016_1001_MOESM3_ESM.docx]

**Table S3 - Allele frequencies of HLA-C types (C1 R80 and C2 K80) in CRC patients and Italian controls.**

| **HLA-c** | **crc**  **(2n=104)** | | **Local controls**  **(2N=116)** | | |
| --- | --- | --- | --- | --- | --- |
| ***HLA-C1 group*** | ***N=*** | ***af%*** | ***N=*** | ***af%*** | ***P value=*** |
| Cw1 | 3 | 2.9 | 6 | 5.2 | NS |
| Cw7 | 29 | 27.9 | 25 | 21.6 | NS |
| Cw8 | 4 | 3.8 | 4 | 3.4 | NS |
| Cw9 (0303) | 2 | 1.9 | 0 | 0 | NS |
| Cw10 (0302,04) | 2 | 1.9 | 3 | 2.6 | NS |
| Cw12 | 13 | 12.5 | 15 | 12.9 | NS |
| Cw14 | 3 | 2.9 | 0 | 0 | NS |
| Cw16 | 6 | 5.8 | 2 | 1.7 | NS |
| **Total C1 alleles** | 62 | **59.6** | 55 | **47.4** | **0.07** |
|  |  |  |  |  |  |
| ***HLA-C2 group*** | ***N=*** | ***af%*** | ***N=*** | ***af%*** | ***P value=*** |
| Cw2 | 3 | 2.9 | 7 | 6.0 | NS |
| Cw4 | 17 | 16.3 | 30 | 25.9 | NS |
| Cw5 | 2 | 1.9 | 6 | 5.2 | NS |
| Cw6 | 14 | 13.5 | 12 | 10.3 | NS |
| Cw15 | 6 | 5.8 | 5 | 4.3 | NS |
| Cw17 | 0 | 0 | 1 | 0.9 | NS |
| **Total C2 alleles** | 42 | **40.4** | 61 | **52.6** | **0.07** |
| C1-C1 | 17 | 32.7 | 15 | 25.9 | NS |
| C1-C2 | 28 | 53.8 | 28 | 48.3 | NS |
| C2-C2 | 7 | 13.5 | 15 | 25.9 | NS |
